# Genetic diversity of *Mycobacterium tuberculosis* clinical isolates in Blantyre, Malawi

**DOI:** 10.1016/j.heliyon.2019.e02638

**Published:** 2019-10-10

**Authors:** Victor Ndhlovu, Anmol Kiran, Derek Sloan, Wilson Mandala, Konstantina Kontogianni, Mercy Kamdolozi, Maxine Caws, Gerry Davies

**Affiliations:** a University of Liverpool, Liverpool, UK; b University of Malawi, College of Medicine, Biomedical Sciences Department, Blantyre, Malawi; c Malawi-Liverpool Wellcome Trust, Blantyre, Malawi; d Academy of Medical Sciences, Malawi University of Science and Technology, Thyolo, Malawi; e Liverpool School of Tropical Medicine, Liverpool, UK; f Birat-Nepal Medical Trust, Kathmandu, Nepal; g University of Saint Andrews, UK; h Edinburgh University, Edinburgh, UK

**Keywords:** Clinical genetics, Genetics, Bioinformatics, Infectious disease, Microbiology, Single molecule real time sequencing, Region of difference, *Mycobacterium tuberculosis*, Single nucleotide polymorphism, Large sequence polymorphisms

## Abstract

Despite the high burden of tuberculosis (TB) worldwide, specific factors influencing disease transmission remain elusive. Long term epidemiological studies and in vitro experimental models provide evidence of variable relative fitness of *Mycobacterium tuberculosis (Mtb)* strains but few such studies are available. Large sequence polymorphisms (LSP) are a robust molecular marker and are feasible as an epidemiological investigative tool. Few *Mtb* molecular epidemiological studies have been reported in Malawi owing to lack of laboratories with molecular tools. We characterized the genetic diversity of *Mtb* clinical isolates amongst TB patients in Blantyre, Malawi. We genotyped 64 Mtb clinical isolates using LSP-PCR, assigned specific lineages and confirmed 18 of the isolates using SMRT sequencing. The 64 isolates clustered into 4 lineages (L1-L4) with L4 predominating. There were 10/64 (16%) isolates belonging to L1, 6/64 (9%) belonging to L2, 2/64 (3%) belonging to L3 and 46/64 (72%) belonging to L4. Comparison with a previous study done in Karonga revealed concordance in L1 and L4 but discodance in L2 and L3. The phylogenetic tree constructed, comprised of 3/4 lineages present in Blantyre with 3/18 belonging to L1, 3/18 belonging to L2 and 12/18 belonging to L4. Four *Mtb* lineages were present in Blantyre with L4 predominating. Larger studies are needed to understand the molecular epidemiology of TB in Blantyre in light of increased bi-directional migration with South Africa.

## 1 Introduction

Globally, there were 10 million new cases and 1.3 million deaths due to tuberculosis (TB) with a further 0.4 million due to coinfection with HIV in 2017 [1]. Africa remains the hardest hit in terms of rates of active TB per capita [2, 3, 4] and the highest proportion of TB cases co-infected with HIV are in Africa (31%) with Southern Africa bearing the greatest burden (>50%) [5].

Malawi is a country in the South East of Africa with a population of about 17.5 million [6] and 24.3% of the population living in severe multi-dimensional poverty [7]. Malawi is among the top 20 high TB burden countries with the highest estimated number of incident TB cases among people living with HIV [4]. Concerning molecular epidemiology of TB in Malawi, a few studies have been reported from the northern region of the country under the Karonga Prevention Study (KPS) [8, 9]. A lack of laboratories with molecular tools has hampered efforts to conduct *Mycobacterium tuberculosis* (*Mtb)* molecular epidemiology studies in Malawi and in most other low resource settings. *Mtb* molecular epidemiological data for the rest of the country is lacking. The currently prevailing *Mtb* lineages for the country remain to be established and longitudinal transmission elucidated. Knowledge of changes in the predominant circulating *Mtb* strains in Blantyre may aid in understanding the local TB epidemic and refining TB control strategies.

In this study we use large sequence polymorphism (LSP) polymerase chain reaction (PCR) to perform a preliminary characterization of the genetic diversity of *Mtb* clinical isolates amongst patients presenting at Queen Elizabeth Central Hospital, Blantyre, Malawi, which is the largest referral hospital in the country. The samples were collected over an 18 months period (June 2010 to December 2011). We used Single Molecule Real Time Sequencing (SMRT) to confirm the phylogenetic lineages of a representative 18 of these strains.

## 2 Materials and methods

### 2.1 Study setting and population

The study was conducted in Blantyre, the largest commercial city in Malawi situated in the southern region of the country. The city has a population of over 800,000 people according to the 2018 population and housing census [6], and 65% of the city's population live in informal settlements with poor living conditions [10].

The study was conducted as part of a larger study “Studying Persistence and Understanding Tuberculosis in Malawi (S.P.U.T.U.M) for which ethical approval had been obtained from the College of Medicine Research Ethics Committee [11] This was a prospective cohort study of persons with smear and culture positive pulmonary TB from Blantyre Malawi. A total of 133 Mtb strains collected from patients presenting with sputum smear positive TB at Queen Elizabeth Central Hospital (QECH) during an 18-month period (June 2010–December 2011) and used for strain typing. Both new and retreatment cases irrespective of HIV status were enrolled and participants were aged 16–65 years. Only 64/133 Mtb strains were successfully revived from frozen state and confirmed as *Mtb*. *Mtb* was confirmed using the BD MGIT TBC ID test device (Becton Dickinson, Maryland U.S.A) and Ziehl Neelsen (ZN) staining and culture on Lowenstein Jensen (LJ) slants following standard laboratory procedures. Bacterial cultures were grown to mid-log phase (6–8 weeks) before being harvested for DNA isolation.

### 2.2 Drug susceptibility testing

Determination of minimum inhibitory concentrations (MICs) of the *Mtb* isolates was carried out using UKMYC3 sensititre custom made plates (Thermo Scientific) following manufacturer's instructions. The plates contained different concentrations of RIF (0.015–16 μg/mL), INH (0.015–16 μg/mL) and EMB (0.25–16 μg/mL). Briefly, a loopful of colonies were scraped from LJ slants and emulsified in a MacFarland tube containing saline, 0.2% tween and glass beads. The tube was vortexed for 30 seconds and turbidity adjusted to 0.5 MacFarland standard. A100 μl aliquot was transferred into a tube of 10ml of 7H9 broth supplemented with 10% OADC to give an inoculum of ∼1 × 10^5^ CFU/mL. The suspension was briefly vortexed and a 100 μl aliquot transferred into each well of the microtitre plate for inoculation. Wells were covered with adhesive seal, wiped with 5% Surfanios and 70% ethanol before being placed into a sealed plastic bag. Plates were incubated at 37 °C for 10 days after which visual inspection of growth was done. Plates with poor growth were re-checked using the positive control at days 14 and 21. Plates were read when growth was clearly visible in the positive control.

### 2.3 DNA extraction and genotyping

Genomic DNA was isolated using a Cetyltrimethylammonium bromide (CTAB) method as previously described [12]. Genotyping of isolates was performed at the Liverpool School of Tropical Medicine, United Kingdom. A singleplex PCR based method using specific primers specific for each of regions of difference RD239 (L1), RD105 (L2) and RD 750 (L3) was employed. Primers were selected based on *Mtb* molecular epidemiology studies in and around Malawi in the last 15 years [13, 14, 15, 16]. All isolates not falling within these three lineages were presumed to be Lineage 4 (Euro-American) and a representative sample were subsequently confirmed by sequencing. PCR reactions were performed in a micro centrifuge tube to a final volume of 15 μL. For an RD239 reaction mixture, to 15ng of DNA was added a PCR master-mix containing 1x buffer (Bioline, UK), 0.2mM dNTPs, 0.5μM each primer (forward and reverse),1.50mM MgCl_2,_ double distilled water and Taq polymerase (Bioline, UK). PCR reactions were performed at 95 °C for one 3-minute cycle, three 25 cycles of 30 seconds at 95 °C, 64 °C and 72 °C respectively and a 6-minute cycle at 72 °C.

For the RD105 and RD 750 the reaction mixture contained 15ng of DNA and a PCR master mix of 1x buffer without MgCl_2_ (EHF), 0.2mM dNTPs, 1.5mM MgCl_2,_ 0.3μM each primer (forward and reverse) and EHF Taq polymerase. PCR reactions were performed at one 3-minute cycle at 95 °C, 25 cycles of 95°C/15 seconds, 64°C/15 seconds, 72°C/4 minutes and 6 minutes at 72 °C. Primers used were RD239 forward F5′-GGC CAA CAT CGA CCA CCT ACC C-3′ and R5′-ATC CTC GCT ACC GGC ACC TCA T-3’. For RD105, F 5′-GGA GTC GTT GAG GGT GTT CAT CAG CTC AGT C-3′ and R 5′-CGC CAA GGC CGC ATA GTC ACG GTC G-3′and for RD750, F5′- GTC AAC TGC CGA TGG CTG AC-3′ and R 5′- CGT CAG CGA TGA TCA CCT CG-3’. PCR products were electrophoresed on 1% agarose, stained with ethidium bromide before being visualized under UV light.

### 2.4 DNA sequencing and bioinformatics analysis

DNA was sequenced at the Centre for Genomic Research (CGR), University of Liverpool, United Kingdom on Pacific Biosciences RSII sequencing system (Pacific Biosciences, Menlo Park, CA, USA). Single Molecule Real Time (SMRT) sequencing was used at 360-minute movie times per cell, yielding ∼174x average genome coverage. All the raw SMRT sequencing data used in this paper have been submitted to the European Nucleotide Archive (ENA) databases under accession no. PRJEB28592 and is accessible at: http://www.ebi.ac.uk/ena/data/view/PRJEB28592. Bioinformatics analysis was conducted using the RS_Modification_and_Motif_Analysis.1 protocol in SMRT Portal (version 2.2.0). Sequence reads were mapped using the Basic Local Alignment with Successive Refinement (BLASR) [17] algorithm within the SMRT portal. A phylogenetic tree was constructed for the 18 sequences using RAxML (Randomized Axelerated Maximum Likelihood) a program for phylogenetic analysis in GTRCAT model [18] based on all polymorphic sites. To analyze methylation patterns in *Mtb* the reference file for H37Rv (GenBank accession NC_000962) containing all genomic annotations in general features format (gff) was downloaded. All rows in the genome containing 6-methyladenine (m6A) modifications were extracted using a custom script run in Python software.

The genomic ranges for the genes were obtained from ProteinTable166_159857.txt (http://www.ncbi.nlm.nih.gov/genome/proteins/166?genome_assembly_id=159857). If the gene was on the positive strand, the start site was reduced by 100 nucleotides while if the gene was on the negative strand, the start site was extended by 100 nucleotides. Any m6A modification found within the gene's genomic range was reported.

## 3 Results

**Fig. 1:**
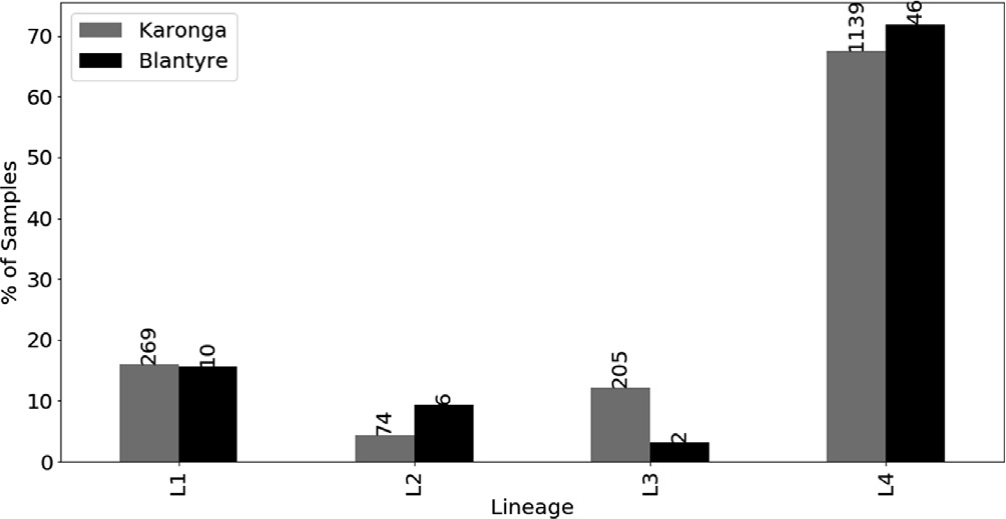
Comparison of the diversity of *Mycobacterium tuberculosis* in Blantyre and Karonga, Malawi.

Sixty-four bacteriologically culture confirmed *Mtb* positive samples were selected based on the first to be successfully revived from a total of 133 isolates and subjected to genomic deletion analysis. The analysis revealed 6 (9%) East Asian lineage (L2) (RD 105 deleted) strains generating a deleted product ∼785bp on gel electrophoresis, 10 (16%) (L1) Indo-oceanic lineage (RD 239 deleted) strains, 2 (3%) East African/Indian lineage (L3) (RD750 deleted) strains and 46 (L4) (72%) Euro-American lineage strains (Fig. 1). No mixed infection was detected as no samples was found with two different deletions.

It was not possible to design lineage specific primers for the Euro-American lineage, a common previously reported lineage in Malawi. Twelve out of the 46 isolates were confirmed as Euro-American using whole genome sequencing. All other strains lacking deletions stated above were assumed to belong to the Euro-American lineage owing to the fact that this lineage was reported to be the most predominant in the region and has previously been reported at a high prevalence in Karonga [13]. Drug sensitivity testing of all the 64 isolates revealed that one isolate was INH resistant and a single isolate was multi-drug resistant (resistant to INH and RIF).

Proportions of *Mtb* lineages occurring in Blantyre; 46 (72%) lineage 4 (Euro-American), 10 (16%) Lineage 1(Indo-oceanic), 6 (9%) lineage 2 (Beijing), 2 (3%) lineage 3 (East African/Indian) and in Karonga *M. tuberculosis* strains (68%) were lineage-4, with 16% lineage-1, 4% lineage-2, and 12% lineage-3 Blantyre samples were collected between June 2010 and December 2012 while Karonga samples were collected between September 1995 and September 2010.

### 3.1 Whole genome sequence analysis

**Fig. 2:**
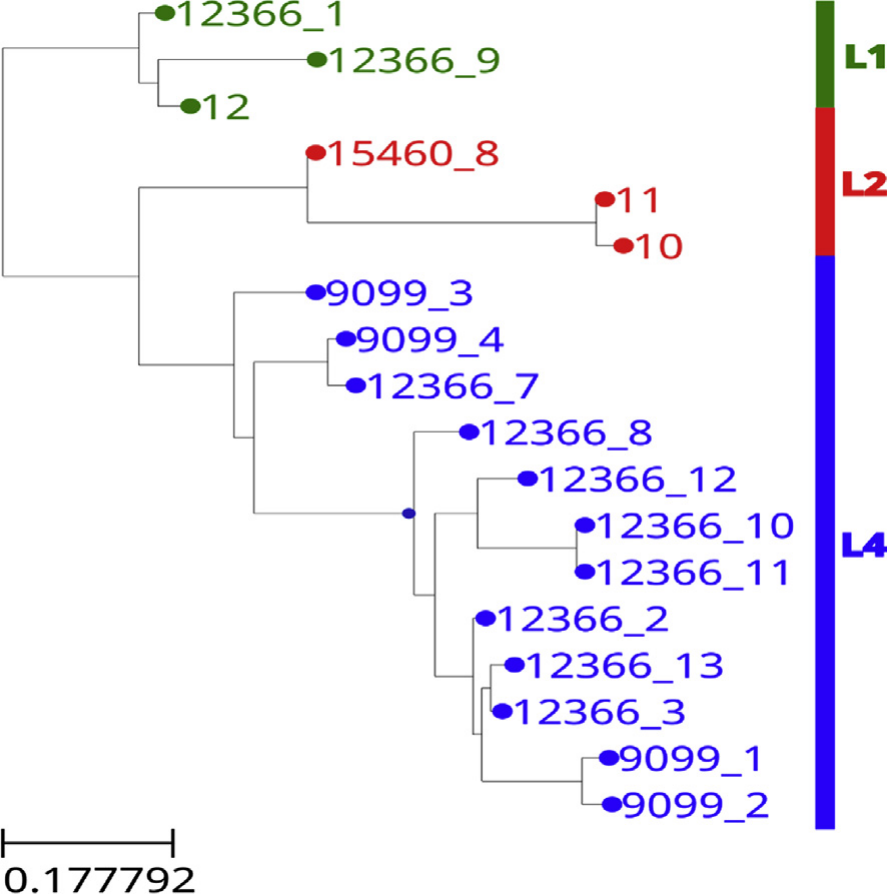
Phylogenetic analysis of the 18 sequenced *Mycobacterium tuberculosis* strains from Blantyre Malawi.

In this study 18/64 Mtb genomes were completed using SMRT sequencing to compare and confirm the results of genomic deletion analysis in this subset of the strains. The average sequencing coverage was ∼x174 (range x35-460) with mean read length ∼8kb (range 3.5–12kb) and N50 read length ∼12kb (range 5–24kb). In comparison with the *Mtb* H37Rv reference genome (NC_000962) [19] the 18 genomes showed >99.8% identity at the nucleotide level. Using phylogenetic analysis, a total of 14,353 SNPs were found in the non-repetitive regions with one sample having 6,909 SNPs not present in any other sample. The SNPs present in more than one sample (7444) were used to construct a genome-wide maximum likelihood phylogenetic tree (Fig. 2) which comprised of 3 of the 4 Mtb lineages known to be present in Malawi (L1, L2 and L4). Three of the 18 strains belonged to lineage 1 (Indo-oceanic), 3/18 to lineage 2 (East-Asian) and 12/18 belonged to lineage 4 (Euro-American). No lineage 3 strain was found among the 18 sequences. All lineage 4 Mtb strains have been reported to possess a 7 bp deletion in the *pks15/1* gene [20]. Screening for this deletion in the 18 strains confirmed that 12/18 strains belonged to lineage 4. Additionally, membership of lineages 1 and 2 was confirmed by the presence of lineage specific mutations causing loss of methyltransferase activity (C758T and A809C respectively) as previously reported [21].

Strains clustered into 3 lineages (L1,2 and 4). Green indicates that strains belong to lineage 1(Indo-oceanic), red indicates that strains belong to lineage 2 (East Asian-Beijing) and blue indicates strains belong to lineage 4 (Euro-American). *M, canettii* was used as an outlier and the branch support values determined by 1000 bootstrap replicates.

## 4 Discussion

This is the first description of *Mtb* genotypes circulating in Blantyre, Malawi. Sixty-four out of one hundred and thirty-three clinical isolates from patients presenting at a referral facility in Blantyre, Malawi were genotyped using genomic deletion analysis and 18 of these compared with classification according to whole genome sequencing. Globally, 7 major lineages of *Mtb* have been defined and 4 of these were detected as circulating in Blantyre. The Euro-American lineage (lineage 4) is the most predominant of the *Mtb* lineages in Europe and America but has specific sub-lineages dominating in Africa [20, 22]. Results in this study and those done previously in Malawi [8, 13] demonstrate complete concordance with those done in this region of Africa [23]. In the present study, lineage 4 was identified by default based on the absence of deletions RD 105, 239 and 750. The fact that no deletion analysis was done to confirm the number of isolates belonging to lineage 4 does not exclude the possibility that a novel lineage not previously reported in Malawi could be present. In addition membership of lineage 4 can be confirmed by the presence of a short 7bp deletion (GCCGCGG) in the polyketide synthase (*pks 15/1)* gene and a CTG to CGG substitution in *katG* gene at position/codon 463 [20, 22, 24, 25]. Bioinformatics analysis of the 18 sequenced isolates in this study revealed the presence of this 7bp deletion in only 12 of the 18 sequences further confirming the findings from the LSP assay. A small sample size not withstanding, our results are largely in concordance with previous studies done in Karonga [8, 9]. In one particular study, out of 781 patients with a first episode of TB, 76% were Euro-American lineage [8] while in another, the lineage was found to occur at 68% [13]. The Euro-American lineage appears to be the predominant lineage in Malawi based on data from both studies although more studies are necessary countrywide to definitively confirm these results and provide greater impact of this lineage on disease burden in Malawi.

The presence of Lineage 1 (16%) detected using PCR in this study is consistent with reports from Karonga [13]. Similarly, phylogenetic analysis confirmed that 3 of the isolates that were identified as lineage 1 (Indo-oceanic) using LSP-PCR were indeed of this lineage. Again, membership of this lineage could further be confirmed by loss of a methyltransferase *mamB* attributed to a missense mutation in the Rv2024c gene [26]. Although lineage 1 has global prevalence, it is more common in East Africa and the Indian sub-continent [27]. Karonga is easily accessible from the East African country of Tanzania which has a higher prevalence of this lineage and so the expectation might have been that a higher prevalence of this lineage would have been observed than in Blantyre. However, a larger study would be needed to draw robust conclusions regarding the prevalence and transmission of L1 throughout Malawi as a whole.

The East Asian lineage (L2) which includes the *Beijing* genotype, has been reported to be widespread globally and may be associated with increasing drug resistance in some regions [28]. In Mozambique, which is close to Blantyre, the modern *Beijing* genotype was associated with increasing virulence compared to ancient strains [29] and also with HIV infection [16]. The results of the published data from Malawi differ in that no association of any lineage with HIV could be established. The phylogenetic tree constructed from SNPs and indels confirmed that 3/18 isolates belonged to L2. Additionally, methylation analysis revealed that these three samples lacked the *mamA* methyltransferase owing to a point mutation in the Rv3263c gene, which is characteristic of L2 clinical isolates [30]. In Malawi the L2 has previously been reported from approximately ∼2% of patients and no association with drug resistance has been reported [8]. All the L2 strains from the current study were drug sensitive which is consistent with the study from Karonga [13]. An association between East Asian lineage/*Beijing* genotype and drug resistance is yet to be seen in Malawi, possibly because the overall prevalence of drug resistance is low by regional standards and L2 remains relatively rare to date. Future studies should investigate genotype-phenotype associations of the L2/*Beijing* genotype in Blantyre and Malawi as a whole. The data from this Blantyre study provides an important baseline to track future emergence of L2/*Beijing* genotype in southern Malawi and any increased association with drug resistance as it emerges. The results presented here may appear to suggest a higher prevalence of the L2 strains at 9% in Blantyre compared to 4% in Karonga [13]. However, it is possible a higher prevalence of the L2 in Blantyre compared to Karonga could be attributed to the fact that Blantyre is an urban setting where transmission rates are likely to be higher. Furthermore Blantyre has a higher geographical accessibility to South Africa and Mozambique where the L2/*Beijing genotype* has been reported at a higher prevalence [16, 31].

A study of strains from histological samples from a 76-year period in South Africa demonstrated that the *Beijing* strain was a recent arrival in the country [14]. It would be reasonable to infer that a similar trend could occur in Malawi as there is a high level of regional migration between Malawi and South Africa. A study from Karonga suggests that the East Asian/Beijing may have only recently arrived in Malawi compared to other *Mycobacterium tuberculosis* lineages [8]. The spread of L2/*Beijing* in Malawi could also be attributed to recent influx of people of Eastern-Asian ethnic background involved in road construction and trade. Additionally, there has been an increase of Malawian business people travelling to China over the past few years. A country-wide study on the diversity of L2/*Beijing* strain in Malawi will be needed to fully confirm the current prevalence of this lineage and its drug sensitivity profile and to monitor trends in its representation among cases of tuberculosis in Malawi in the future. In this study we have demonstrated concordance in lineage assignment of strains using LSP-PCR, SNP analysis and DNA methylation analysis. We have shown that the circulating *Mtb* strains in Blantyre, southern Malawi, are broadly similar to those previously described in northern Malawi [13]. The L2 and drug resistance remain rare in both locations, with Euro-American (L4) strains predominating. The increasing bidirectional economic migration between Malawi and China, may lead to changes in the molecular epidemiology of *Mtb* in Malawi over the coming decade and this study provides a first baseline insight into the current pattern of circulating *Mtb* strains. Overall the assay used in this study did not detect any mixed infections. Presence of mixed infections would have meant overrepresentation of certain strains thereby given different results. A more robust method such as SNP typing or spoligotyping would be required to give a greater insight of the circulating strains in Blantyre.

## Declarations

### Author contribution statement

V. Ndhlovu: Conceived and designed the experiments; Performed the experiments; Analyzed and interpreted the data; Wrote the paper.

A. Kiran: Analyzed and interpreted the data; Wrote the paper.

D. Sloan: Contributed reagents, materials, analysis tools or data; Wrote the paper.

W. Madala: Contributed reagents, materials, analysis tools or data; Analyzed and interpreted the data.

K. Kontogianni, M. Kamdolozi: Performed the experiments.

M. Caws: Conceived and designed the experiments; Wrote the paper.

G. Davies: Conceived and designed the experiments, Contributed reagents, materials, analysis tools or data; Wrote the paper.

### Funding statement

This work was supported by The Helse Nord Tuberculosis Initiative (HNTI).

### Competing interest statement

The authors declare no conflict of interest.

### Additional information

Data associated with this study has been deposited at European Nucleotide Archive under the accession number PRJEB28592.
